# Endoscopic Closure for EUS and ERCP Related Duodenal Perforation by Endoclips

**DOI:** 10.1155/2016/1051597

**Published:** 2016-09-15

**Authors:** Yaping Liu, Dong Wang, Zhaoshen Li

**Affiliations:** Department of Gastroenterology, Changhai Hospital, The Second Military Medical University, Shanghai 200433, China

## Abstract

*Objective*. To investigate the therapeutic safety, feasibility, and efficacy of endoclips for closing the endoscopic ultrasound (EUS) and endoscopic retrograde cholangiopancreatography (ERCP) related duodenal perforation in a retrospective study from a single center.* Methods*. Patients who developed EUS and ERCP related duodenal perforation between January 2012 and January 2015 were included in the study. All the cases underwent endoscopic closure by endoclips, and the efficacy, feasibility, and safety of this technique were evaluated.* Results*. During the study period, a total of 17,406 patients were treated by EUS and/or ERCP. EUS and ERCP related duodenal perforation occurred in 9 cases (0.05%): 2 males and 7 females. The mean age was 69 years (range: 59–79 years). The success rate of endoscopic closure by endoclips was 100%. The mean procedure time was 45 ± 12.5 min. The mean number of endoclips placed for the closure of the duodenal perforation was 7 ± 3.2. All the patients recovered completely without any severe complications.* Conclusion*. The endoscopic closure by using endoclips is recommended as the first-line treatment for duodenal perforation associated with EUS and ERCP.

## 1. Introduction

Endoscopic ultrasound (EUS) and endoscopic retrograde cholangiopancreatography (ERCP) are widely used for the diagnosis and treatment of various pancreatic and biliary diseases [1]. Although the endoscopic techniques of EUS and ERCP have greatly improved over the years, the high incidence of procedure-related complications still remains a challenge [2]. Prevention and prompt treatment of complications are of vital importance to further expand the usage and improve the effectiveness of EUS and ERCP.

Duodenal perforation is one of the severe complications associated with EUS and ERCP [3, 4]. Patients diagnosed with duodenal perforation may progress to acute peritonitis and septic shock, which is associated with a high mortality rate [5]. Open surgery has been the traditional therapeutic method for years. In 1997, Yoshikane et al. first reported the endoscopic closure by using endoclips in a patient with duodenal perforation [6]. The endoscopic closure by using endoclips has the advantages of being effective, being minimally invasive, and having a shorter recovery time, which makes it an ideal treatment modality for gastrointestinal perforation, especially duodenal perforation [4, 7]. So far, few large-scale studies have been conducted to verify the clinical significance of this technique. In order to better understand the therapeutic potential of this endoscopic intervention, we conducted this retrospective study to determine the therapeutic safety, feasibility, and efficacy of endoclips for closure of duodenal perforation.

## 2. Methods

### 2.1. Patients

Data of patients who underwent EUS and/or ERCP from January 2012 to January 2015 at the Endoscopy Center of Changhai Hospital was retrospectively retrieved from the computerized database. The medical records were reviewed and those patients diagnosed with duodenal perforation were included in this study. This study was approved by the Ethics Committee of Changhai Hospital. Informed consent was obtained from all the patients.

### 2.2. ERCP and EUS

All the endoscopic interventions were performed by experienced endoscopists using standard endoscopes. The indications included unknown abdominal pain and suspected pancreatic cancer and common bile duct stone. The diagnosis of duodenal perforation was made on the basis of the endoscopic findings and the clinical manifestations. All the patients underwent computed tomography to confirm the diagnosis of perforation.

### 2.3. Endoscopic Closure by Endoclips

**Figure 1:**
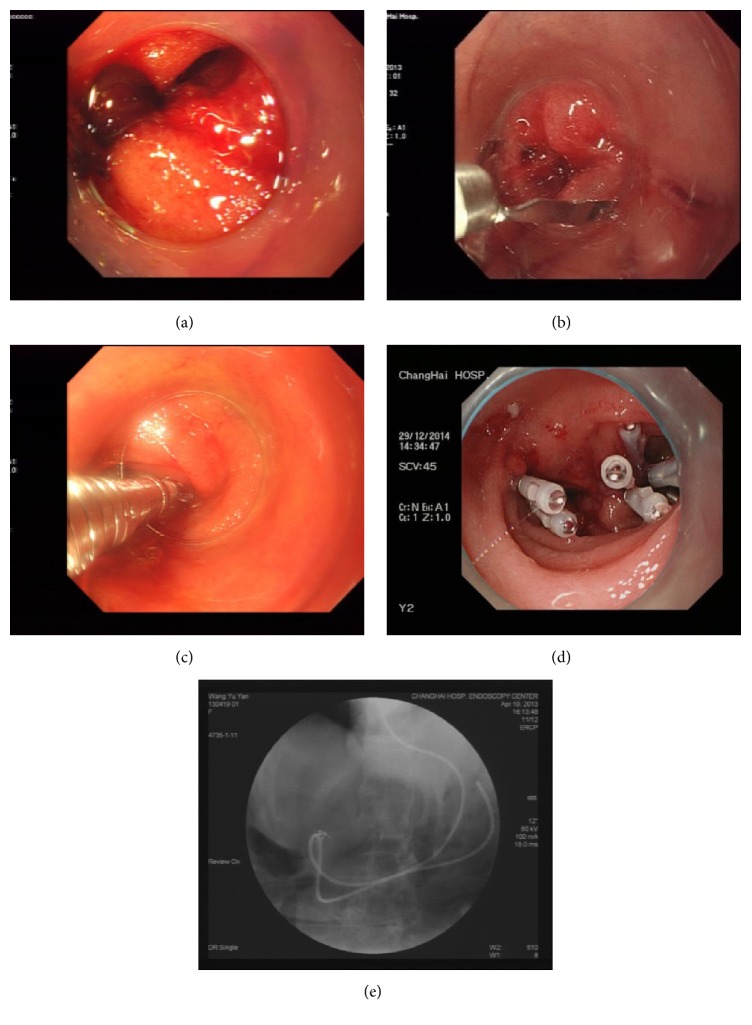
Endoscopic closure by endoclips. The perforation was completely exposed (a). Then, the endoclip was applied to close the perforation (b, c), and the procedure was repeated if the perforation was large in size (d). After the procedure, a nasogastric tube was placed near the perforation site (e).

**Figure 2:**
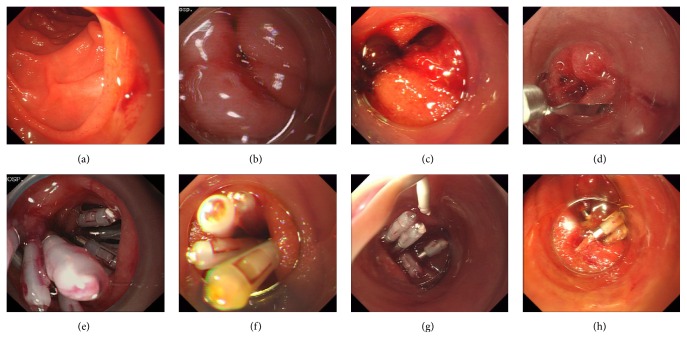
Typical endoscopic images were shown before (a–d) and after (e–h) endoclipping.

Once the duodenal perforation was detected, a transparent capsule was applied to help expose the lesion (Figure 1(a)). The endoclips, including Long Clip (Olympus, Japan) and Resolution® Clip (Boston Scientific, USA), were adjusted to ensure that the entire perforation was within the closure range of the endoclips (Figure 1(b)). Then, the endoclips were released (Figure 1(c)). If the perforation was large in size, multiple endoclips were applied one after the other (Figure 1(d)). After the procedure, the patients were placed in a semireclining position and closely monitored for 48 h. The patients were fed through the nasogastric tube for a period of 2-3 days (Figure 1(e)). Typical endoscopic images were shown in Figure 2. Anastaltic (ethamsylate and para-aminomethylbenzoic acid injection) and antacid treatment was given along with nutritional support and prophylactic antibiotics were administered. The clinical symptoms and vital signs were recorded. All the patients were followed up at one week and at one month after the procedure. Patients judged their satisfaction with the procedure as satisfied and unsatisfied. Our procedure was in accordance with our general local policy protocol aligned with ESGE guidelines.

## 3. Results

### 3.1. Demographic and Clinical Characteristics

A total of 17,406 patients underwent EUS and/or ERCP at our endoscopic center. Duodenal perforation was diagnosed in 9 patients (0.05%): 7 females and 2 males. The mean age was 69 years (range: 59–79 years). The main complaints included fever, abdominal pain, and jaundice. The details of the demographic and clinical characteristics are shown in Table 1.

Three cases of duodenal perforation occurred during EUS and six cases occurred during ERCP. All the three cases of EUS related duodenal perforation were caused by mechanical injury to the duodenal wall by the endoscope. Among the 6 cases of ERCP related perforation, two patients had a previous history of Billroth II subtotal gastrectomy and the perforation site was in the afferent loop. The other four patients had duodenal perforation in the posterior wall of the descending duodenum, which resulted from the extreme bending of the endoscope body.

### 3.2. Analysis of the Efficacy and Safety of Endoscopic Closure by Endoclips

The success rate of endoscopic closure by endoclips was 100% (Table 2). The mean procedure time was 45 ± 12.5 min. The mean number of endoclips required for closing the duodenal perforation was 7 ± 3.2. All the patients complained of transient abdominal pain and symptoms in 88.9% of the patients were alleviated within 24–48 h. The mean duration of hospitalization was 3 ± 0.5 days. No secondary bleeding, perforation, or abdominal infection was detected and there were no severe complications, including death, after the procedure. All the patients recovered completely. Abdominal CT after 1 week of the procedure confirmed that the endoclips were in place, with absence of any gas and fluid in the abdominal cavity. Repeat duodenoscopy after 1 month showed that the perforated area had healed completely with endoclips still in place.

## 4. Discussion

With a wide application of endoscopic interventions in clinical practice, the incidence of iatrogenic perforation is steadily increasing. Carrara et al. reported that duodenal perforation occurred in 0.09% of the 3,296 patients who underwent EUS-fine needle aspiration [8]. The incidence of ERCP related duodenal perforation has been reported to be 0.3–1% [9] and even higher in patients with previous Billroth II subtotal gastrectomy. In order to prevent severe complications and mortality, it is important to make an early diagnosis and start timely treatment [10].

Patients diagnosed with duodenal perforation can be efficiently treated by emergency surgery, but it has the disadvantages of being invasive, having high complication risk, and having a high cost. Thus, endoscopic closure of duodenal perforation might be a better therapeutic option for such patients. Recently, the endoscopic closure by endoclips has become one of the standard treatments for gastrointestinal perforation [11, 12]. However, due to its low incidence, very few case reports on duodenal perforation have been published. von Renteln et al. compared the therapeutic efficacy of surgery and endoscopic closure of duodenal perforation in a pig model [13], which showed that the results of the latter were comparable to surgery, while being more feasible. Mangiavillano et al. reported successful treatment of a patient with EUS related duodenal perforation by using endoclip [14]. In this study, we successfully treated all the 9 patients by using endoclips.

We also summarized our experience, which might help establish the standard protocol for the management of such patients. First, side-viewing and oblique-viewing endoscopy should be replaced by forward endoscopy with transparent capsule and carbon dioxide infusion, which has more flexibility and exposes the perforation completely. As described earlier, in our endoscopic center, we used MH-463 (Olympus, Japan) combined with HX-610-135L (Olympus, Japan) which showed no severe complications during the entire study period. Second, for large perforations, multiple endoclips should be placed and endoloops could also be applied when necessary. Long Clip (Olympus, Japan), Resolution Clip (Boston Scientific, USA), Tri-Clip and Instinct Clip (Cool, USA), and Over-the-Scope Clip (OTSC, Ovesco, Germany) are generally applied in clinical practice. In this study, the endoscopic closure by Long Clip and Resolution Clip was successful and curative. Recent studies recommend the use of an endoclipping device for the management of iatrogenic gastrointestinal perforations in select cases that fulfill the following criteria: instant identification of the perforation during the procedure; a tear that is less than 10 mm in size; an endoscopy team that is experienced with using endoclips; and the availability of surgical help if necessary [15]. In our study, we successfully treated one patient with perforation that was over 10 mm in size. The total number of endoclips must not be limited, and these endoclips are excreted after the healing of the perforation. Third, after the procedure, nasogastric decompression should be administrated, which helps quicken the recovery of the mucosa and minimize the injury by gastric acid. Fourth, for the patients with obstructive icterus, ERCP should be stopped and percutaneous transhepatic cholangial drainage should be performed to drain the bile duct. Then, after one week, ERCP could be repeated. CT examination should be conducted before and after the procedure in order to evaluate the severity of the disease and further guide the therapeutic strategy for the patients. Finally, oral administration of a contrast agent may help to ascertain whether the perforation is healed or not.

The endoscopic placement technique of clips in the lateral wall of the duodenum is still challenging due to the relatively high complication incidence. The perforation usually occurs in the posterior wall, upper corner, and descending segment of the duodenum, which could not be clearly observed by side-viewing endoscopy when ERCP or EUS is conducted. This may be caused by the following reasons: (1) the space in the duodenal bulb was quite limited and the technique difficulty is high and (2) it is very difficult to stabilize the endoscopy in the descending duodenum due to the spaciousness of the stomach. The function of the transparent capsule is to both expose the lesion completely and protect the mucosa from being injured by endoclips because the endoclips are placed after accurately localizing the perforation and the process of localization may injure the mucosa. In addition, we change the side-viewing endoscopy for forward-viewing endoscopy used once the perforation is detected.

Taken together, our results indicate that endoscopic closure by endoclips is a safe, feasible, and effective technique for the treatment of EUS and ERCP related duodenal perforation. However, the findings of this study need to be further validated by large-scale multicenter clinical trials, due to the limitation of enrolling a small sample from a single center.

## Figures and Tables

**Table 1:** Demographic and clinical characteristics of the patients (*n* = 9) with duodenal perforation.

Case number	Age (years)	Gender	Main complaint	Endoscopic diagnosis
1	60	Female	Abdominal pain	Mild common bile duct dilation
2	63	Female	Abdominal pain and jaundice of skin and sclera	Duodenal diverticulum and pancreatic cancer
3	74	Female	Intermittent abdominal pain	Duodenal mucosal laceration and mass in common bile duct
4	76	Female	Abdominal pain	Mass in duodenal papilla
5	79	Female	Jaundice of skin and sclera	Duodenal diverticulum
6	72	Female	Abdominal pain	Duodenal ulcer and common bile duct stone
7	77	Female	Epigastric pain and jaundice of skin and sclera	Pancreatic cancer
8	59	Male	Intermittent abdominal pain	Common bile duct stone
9	77	Male	Fever and jaundice of skin and sclera	Common bile duct stone

**Table 2:** Endoscopic closure of the duodenal perforation.

Case number	Perforation site	Diameter (mm)	Endoscopic closure	Therapeutic efficacy	Outcome	Number of endoloops placed
1	Greater curve of duodenal bulb	8 × 6	Endoclips and endoloops	Complete remission	Good	7
2	Posterior wall of descending duodenum	5 × 4	Endoclips	Complete remission	Good	3
3	Upper corner of duodenum	6 × 5	Endoclips	Complete remission	Good	5
4	Descending duodenum	12 × 8	Endoclips	Complete remission	Good	8
5	Lateral wall of descending duodenum	7 × 5	Endoclips	Complete remission	Good	6
6	Posterior wall of descending duodenum	20 × 20	Endoclips	Complete remission	Good	12
7	Descending duodenum	10 × 10	Endoclips	Complete remission	Good	10
8	Descending duodenum	8 × 6	Endoclips	Complete remission	Good	7
9	Descending duodenum	5 × 3	Endoclips and endoloops	Complete remission	Good	3
